# RAC1 Involves in the Radioresistance by Mediating Epithelial-Mesenchymal Transition in Lung Cancer

**DOI:** 10.3389/fonc.2020.00649

**Published:** 2020-04-28

**Authors:** Shiming Tan, Pin Yi, Heran Wang, Longzheng Xia, Yaqian Han, Hui Wang, Biao Zeng, Lu Tang, Qing Pan, Yutong Tian, Shan Rao, Linda Oyang, Jiaxin Liang, Jinguan Lin, Min Su, Yingrui Shi, Qianjin Liao, Yujuan Zhou

**Affiliations:** ^1^Hunan Key Laboratory of Translational Radiation Oncology, Hunan Cancer Hospital and The Affiliated Cancer Hospital of Xiangya School of Medicine, Central South University, Changsha, China; ^2^Hunan Cancer Hospital, University of South China, Hengyang, China; ^3^Hepatology Unit, Department of Infectious Disease, Nanfang Hospital, Southern Medical University, Guangzhou, China

**Keywords:** RAC1, lung cancer, radioresistance, epithelial-to-mesenchymal transition, metastasis

## Abstract

Radiation therapy is a common and acceptable approach for lung cancer. Although the benefit of ionizing radiation (IR) is well-established, cancer cells can still survive via pro-survival and metastasis signaling pathways. Ras related C3 botulinum toxin substrate1 (RAC1), a member of Rho family GTPases, plays important roles in cell migration and survival. In the present study, we investigated the effects of RAC1 on the survival of lung cancer cells treated with irradiation. The results showed RAC1 is overexpressed in lung cancer cells and promoted cell proliferation and survival. Furthermore, IR induced RAC1 expression and activity via the activation of PI3K/AKT signaling pathway, and then enhancing cell proliferation, survival, migration and metastasis and increasing levels of epithelial-to-mesenchymal transition (EMT) markers, which facilitated the cell survival and invasive phenotypes. In addition, overexpression of RAC1 attenuated the efficacy of irradiation, while inhibition of RAC1 enhanced sensitivity of irradiation in xenograft tumors *in vivo*. Collectively, we further found that RAC1 enhanced radioresistance by promoting EMT via targeting the PAK1-LIMK1-Cofilins signaling in lung cancer. Our finding provides the evidences to explore RAC1 as a therapeutic target for radioresistant lung cancer cells.

## Introduction

Lung cancer is the most common cancer and one of the leading causes of death worldwide (1). The majority of lung cancer is NSCLC (non-small cell lung cancer) includes squamous cell carcinoma, adenocarcinoma, and large cell carcinoma (2). Medical treatments to cure NSCLC are mainly surgery, radiation therapy, and chemotherapy (3). Radiation therapy combined with chemotherapy can produce a cure only in a small number of NSCLC patients because NSCLC is comparatively less sensitive to chemotherapy or radiation (4, 5). Acquired resistance results in local tumor recurrence or failure of radiotherapy (6, 7). Increasing evidence has demonstrated that epithelial-to-mesenchymal transition (EMT) mediated tumor metastasis is closely associated with radiation resistance (8, 9). The biological process during EMT endows epithelial cells lose their apical-basal polarity and acquire mesenchymal cell traits (10, 11), which is characterized by the loss of epithelial morphology and the acquisition of mesenchymal morphology, of which the epithelial markers includes E-cadherin, Desmoplakin, Occludins, Claudins, and ZO-1) and mesenchymalmarkers includs N-cadherin, vimentin, and FSP1. Moreover, the activation of Wnt, TGF-β, and NF-κB signaling pathway are validated in this process (12–14). Many radioresistant cancer cells demonstrate EMT is considered to link to adaptation to hypoxia (15), enhanced DNA repair capacity (10, 15, 16), activated molecular events, and signaling pathways (17).

Ionizing radiation (IR) therapy is routinely used for lung cancer treatment (9, 18). IR could induce DNA damage in cancer cells and subsequently lead to cell death. However, the survival of cancer cells with radioresistance to IR remained a major problem in radiotherapy (19). Thus, it is important to improve understanding of the mechanisms that cancer cells bear IR-induced cytotoxicity. It is well-established that several pathway are cancer cells activated to promote DNA repair and cell survival in response to IR (20), such as cell cycle checkpoint activation, DNA repair pathway, ATM/ATR, PI3K/AKT, and MEK/ERK activation, which promotes survival of cancer cells through up-regulation of anti-apoptotic factors and down-regulation of pro-apoptotic factors (12, 21–24). In addition, The changes in the tumor microenvironment (TME) induced by IR promote tumor invasion and metastasis, which is accompanied with EMT occurrence (25–28). Therefore, it is necessary to explore the relationship of IR and EMT and uncover the potential mechanisms of radioresistance.

Ras-related C3 botulinum toxin substrate 1 (RAC1), as a member of the Rho family of small guanosine triphosphatases (GTPases), has been shown to play a critical role in cytoskeleton reorganization, cell migration and cell survival (29). RAC1 has been found to be overexpressed in various tumors, and is closely associated with tumor migration, invasion and poor prognosis of carcinomas (30). Down-regulated of RAC1 expression or loss of its function significantly suppressed cancer cell proliferation and metastasis (31). Recent study found RAC1 pathway was activated in the regulation of breast cancer cell response to IR is to induce G_2_/M checkpoint activation (32). In contrast, inhibition of RAC1 GTPase sensitizes to γ-irradiation in pancreatic cancer cells (33). However, the role of RAC1 in lung cancer radiotherapy is poorly understood. In this study, the function of RAC1 in human lung cancer cells and its role in response to IR were investigated.

## Materials and Methods

### Cell Culture and Treatment

A549, PC9, H1299, H460, and HBE cells were maintained in RPMI-1640 medium supplemented with 10% FBS and 100 U/ml penicillin-streptomycin solution (Bioind, Israel) in 5% CO_2_ incubator. CMV-RAC1, CMV-sh-RAC1 and their controls plasmids were purchased from Shanghai Genechem Co., LTD. A549 and PC9 Cells transfected using Lipofectamine™3000 (Invitrogen, Waltham City, Massachusetts, USA) with vector, RAC1, sh-control and sh-RAC1 plasmids followed by 500 μg/ml G418 screening were pooled collected and positive clones were selected and identified for stably expressing RAC1 or silencing RAC1. For experiments involving IR exposure, growing cells with serum-free culture medium after indicated dose of 2 Gy/min were continuously incubated with 10% FBS at 37°C for 48 h prior to analysis.

### Antibodies

Anti-E-cadherin (ab15148), anti-Vimentin (ab92547), anti-N-cadherin (ab98952), anti-RAC1 (ab33186), anti-PAK-1 (ab40582), anti-phospho-PAK-1 (ab40795), anti phospho-LIMK1 (ab31341), anti-Ki67 (ab15580), and anti-Caspase-3 (ab13847) was from Abcam (London, UK). Anti-LIMK1 (G2241-2E9) was from Abnova (Taipei, China). Anti-Cofilin1 (#5175) and anti-phospho-Cofilin1 (#3311) were from Cell Signaling Technology (Danvers, MA, USA); anti-GAPDH (60004-1-Ig) was from Proteintech (Chicago, Illinois, USA). HRP-labeled goat anti-rabbit IgG and HRP-labeled goat anti-mouse IgG were from Beyotime (Shanghai, China).

### Establishment of Stably Rac1-Overexpression/Interfering Cell Lines

PCMV-Rac1 and PCMV sh-RAC1 interfering plasmids were used to obtain A549 and PC9 stable expressing RAC1 or stable down-regulated RAC1 according to the protocol in our previous study (34). The target sequences of scramble and shRAC1 were 5′-TTCTCCGAACGTGTCACGT-3′ and 5′-TGCAGTAGATGATGAAAGAAA-3′, respectively.

### Cell Proliferation Assay

A549 and PC9 cells numbers was measured as previously described (35). Briefly, cells were then seeded at 96-well microplates at a density of 3 × 10^3^ cells/well. CCK-8 assay was performed to assess the viability according to the manufacturer's protocol. The experiment was performed in triplicate.

### Clone Formation Assay

Clone formation assay was performed as described previously (36). Briefly, 300 cells were seed at 6-well microplates and incubated for 10–14 days until colonies formed. For IR-induced experiment, different numbers of cells were exposed to IR at an indicated dose of 0, 2, 4, 6, 8 Gy (37) and the colonies were visualized by crystal violet staining and quantified using Image J software.

### Wound-Healing Assay

Cells were seeded into 6-well plates and cultured up to 80% cell density (Corning, Corning City, State of New York, USA). The artificial “wound” was scratched by a 10 μl pipette tip and culture with serum free culture medium. Measure the gap size followed by photographing using an inverted microscope.

### Transwell Migration and Invasion Assays

Transwell migration and invasion assays were performed as described previously (36). The cells were stained with crystal violet and counted under a microscope (Olympus Corp, Tokyo, Japan). The results were statistically analyzed.

### Western Blot Analysis

Western blotting was performed as described previously (36). The blots were visualized using Immobilon Western Chemiluminescent HRP Substrate (EMD Millipore). Images of the western blotting products were captured and analyzed using Quantity One V4.31 (Bio-Rad Laboratories, Inc.).

### RAC1 Pull-Down Assays

Rac1 pulldown assay was performed using commercial kit (#8815, CST) as described recently (36). RAC1-GTP was detected by western blotting using antibodies specific for RAC1 (1:100, CST).

### Quantitative Real-Time PCR (qRT-PCR)

Total RNA was extracted from the cells with TRIzol (Invitrogen; Thermo Fisher Scientific, Inc.) and total RNA (1 μg) was used to synthesize cDNA using (cDNA synthesis kit Thermo Fisher Scientific, Inc.). The reaction system was applied according to the manufacturer's protocol: 42°C for 15 min and 95°C for 3 min. The PCR primers (GAPDH, forward 5′-AGCGAGCATCCCCCAAAGTT-3′, reverse 5′-GGGCACGAAGGCTCATCATT-3′; RAC1, forward 5′-CCCTATCCTATCCGCAAACA-3′, reverse 5′-CGCACCTCAGGATACCACTT-3′; and E-cadherin, forward 5′-ACTGGGACGACGACATGGAAAAG-3′ and reverse 5′-TAGATGGGGACATTGTGGGT-3′) were designed and validated by Sangon Biotech Co., Ltd. (Shanghai, China). qPCR was performed at 95°C for 5 min, followed by 40 cycles of amplification at 95°C for 10 s and 60°C for 30 s on an Applied Biosystems 7500 Real-Time PCR system (Applied Biosystems; Thermo Fisher Scientific, Inc.). The fluorescent signals were collected during the extension phase, quantification cycle (Cq) values of the sample were calculated, and transcript levels were analyzed using the 2–ΔΔCq method. The results were normalized to GAPDH.

### Immunohistochemistry (IHC)

Immunohistochemistry were performed as previously described (36). The specimens were paraffin embedded, and the paraffin sections were subject to antigen retrieval by microwave irradiation in citrate buffer, then treated with 0.3% H_2_O_2_, PBS (pH 7.35–7.45) and further blocked in 3% BSA/PBS. After hydration, the paraffin sections were executed according to the immunohistochemical kit (Cwbiotech Company, Beijing, China) protocols. Anti-Rac1 (1: 150), anti-Ki67 (1:500), and anti-caspase3 (1:150) were used as the primary antibodies. The samples were scored according to the staining intensity and number of positive cells as described previously (38). (1) staining intensity, no observed cell staining was scored as 0, cells with weak staining as 1, cells with moderate staining as 2, cells with moderate staining as 3; (2) number of positive cells, no positive cells were scored as 0, <25% of positive cells as 1, between 25 and 50% positive cells as 2, positive cells over 50% as 3. Next, the score was obtained by multiplying the intensity and reactivity rates. Scores of <4 suggested low expressions, and the remainder were classified as high expression. The immunostaining results were confirmed independently by two pathologists in a blinded manner.

### Animal Experiments

Animal experiment was also performed as previously described (36). Briefly, a total of 5 × 10^6^ cells were subcutaneously injected into the nude mice. The tumor sizes were measured with calipers every other day. Tumor size was estimated by the modified ellipsoid formula: 1/2 (length × width^2^). When the tumor size reached up to ~120 mm^3^, tumors were irradiated with a dose of 15 Gy (5 Gy × 3 fractions). Body weights and tumor volumes were measured every other day. Tumor volumes were calculated by measuring the length [L] and width [W] of tumors using calipers. The formula tumor volume = (L × W^2^)/2 was used to calculate the tumor volume. This experiment was conducted in accordance with the National Institutes of Health Guide for the Care and Use of Laboratory Animals and approved by the Institutional Animal Care and Use Committee of the Hunan Cancer Hospital and The Affiliated Cancer Hospital of Xiangya School of Medicine, Central South University (Changsha, China).

### Statistical Analysis

All statistical analyses were conducted using PRISM GraphPad 7.0. Statistical analyses were performed by means of the Student's *t*-test and the Mann-Whitney *U* test. Data are presented as the mean ± standard deviation. *P* < 0.05 was considered to indicate a statistically significant difference.

## Results

### RAC1 Regulates Cell Proliferation in Lung Cancer Cells *in vivo* and *in vitro*

**Figure 1:**
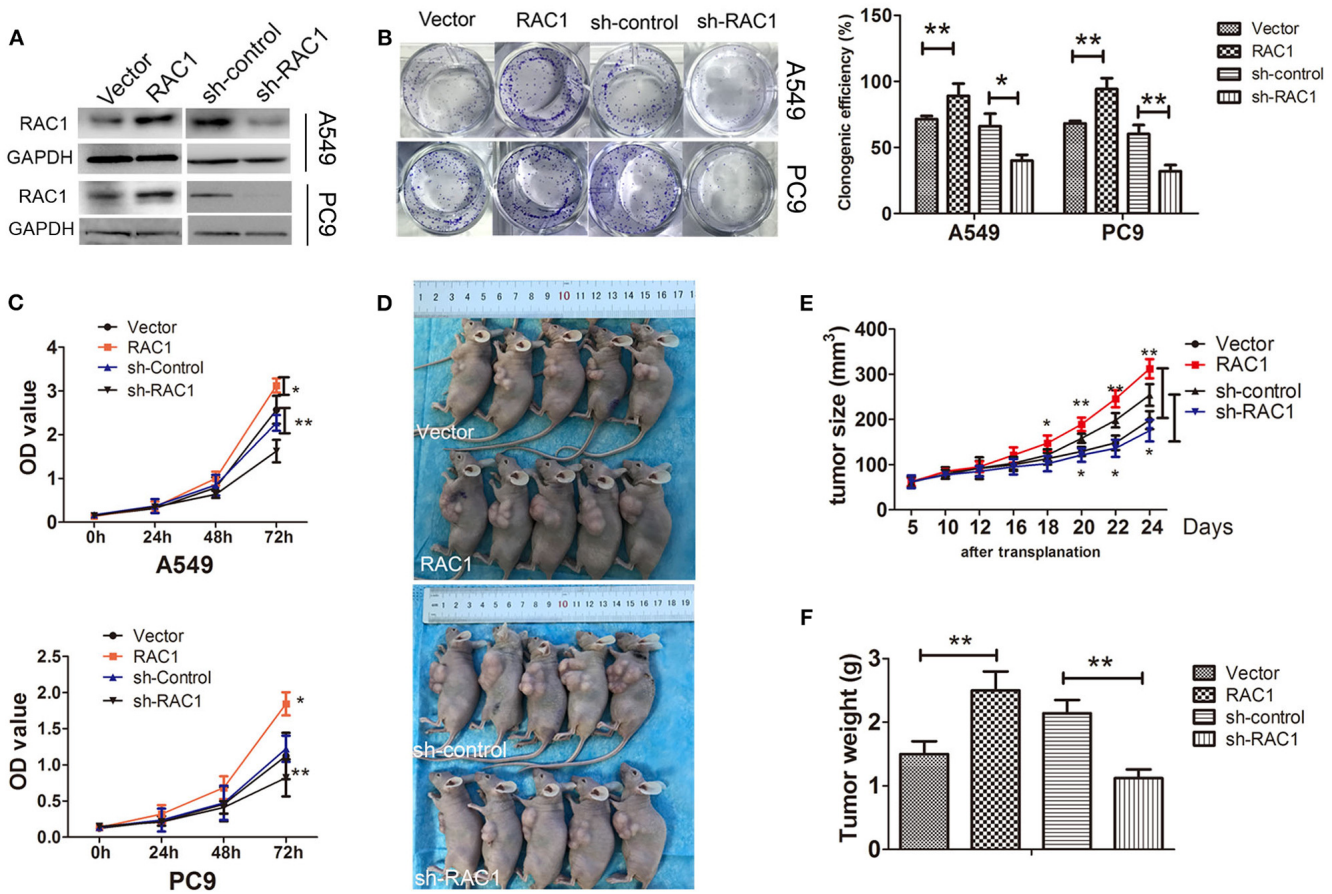
RAC1 regulates cell proliferation *in vivo* and *in vitro* in lung cancer cells. **(A)** The successful overexpression/downregulation of RAC1 protein in A549 and PC9 cells was detected by immunoblotting. **(B)** Overexpression of RAC1 promoted A549 and PC9 cell clone formation capability and silence of RAC1 inhibited cell clone formation capability, which were analyzed by colony formation assay and crystal violet staining after 14 days, clone numbers were quantified. **(C)** The effect of RAC1 expression onA549 and PC9 cell proliferation was assessed by the CCK-8 cell growth assay. A549 and PC9 cells transfected with CMV-RAC1 or CMV-sh-RAC1 plasmid, Vector cells transfected with CMV plasmid or CMV-sh-control plasmid. **(D–F)** RAC1 expression increased tumor growth *in vivo*. The subcutaneous transplantation tumor model in nude BALB/c mice was established using A549 cells that were stably transduced with RAC1 and sh-RAC1. The tumor size was measured at pointed time and tumor weight was measured. Data are expressed as the mean ± SD of different groups of cells from three separate experiments.^*^P < 0.05, ^**^P < 0.01.

Overexpression of RAC1 has been detected in the great majority of lung cancers (39). Our previous study have been elucidated that RAC1 expression is correlated with the expression of EMT markers in non-small cell lung cancer (NSCLC) (38). We first analyzed RAC1 mRNA and protein expression in normal lung cell (HBE) and four NSCLC cell lines (H460, H1299, A549, and PC9). The results showed that RAC1 expression was elevated in four NSCLC cell lines relative to normal lung HBE epithelial cells (Figures S1A,B). We then examined the biological function of RAC1 in lung cancer cells. PC9 and A549 cells with stable RAC1 or sh-RAC1 transfection had moderate RAC1 expression levels (Figure 1A and Figure S1C). The colony formation assays showed that ectopic overexpression of RAC1 promoted growth of both A549 and PC9 cells compared to empty vector control (Figure 1B). In contrast, inhibition of RAC1 decreased the ability of colony formation (Figure 1B). Furthermore, CCK-8 assays demonstrated that overexpression of RAC1 promoted cell proliferation, but silencing of RAC1 expression inhibited cell proliferation in both A549 and PC9 cells (Figure 1C). Tumor increased at a much rapid rate in nude mice in the RAC1 compared with the control group (*P* < 0.05) (Figures 1D,E), while tumor weight was significantly larger in the RAC1 group (Figure 1F). On the other hand, tumor increased at a lower rate in nude mice in the sh-RAC1 compared with sh-control group, and tumor weight was smaller in the sh-RAC1 group (Figures 1D,E). These results suggest that RAC1 promotes proliferation of lung cancer cells.

### IR Induces RAC1 Expression and EMT in Lung Cancer Cells

**Figure 2:**
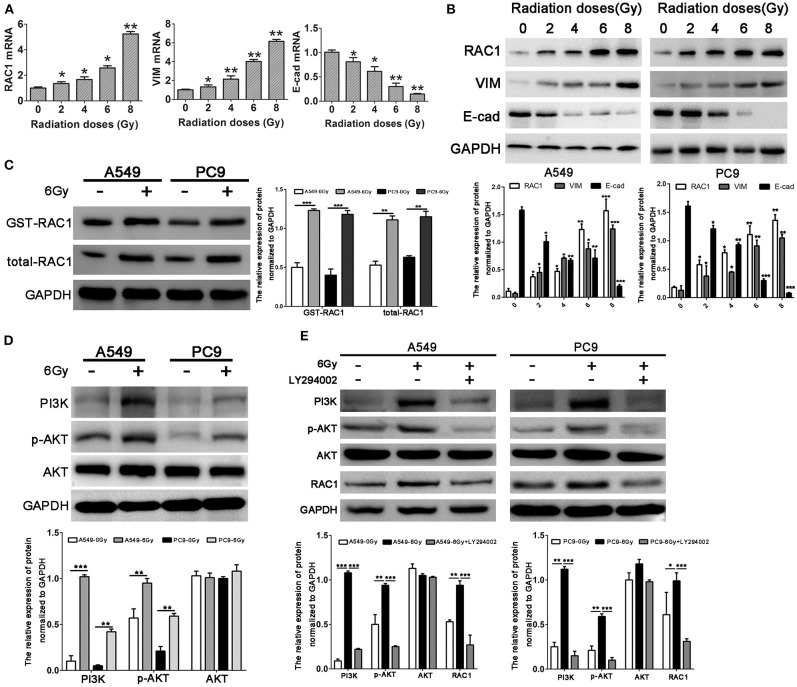
Increased RAC1 expression by irradiation is closely related to EMT markers expression in lung cancer cells. **(A,B)** mRNA and protein levels of RAC1, Vimentin and E-cadherin in A549 cells exposure to 0, 2, 4, 6, 8 Gy dose of irradiation. **(C)** Rac1 expression and activity was monitored in A549 and PC9 cells exposure to 6 Gy dose of irradiation. **(D)** The protein levels of RAC1, PI3K, p-AKT, AKT in A549 and PC9 cells exposure to 6 Gy dose of irradiation. **(E)** The protein levels of RAC1, PI3K, p-AKT, AKT in A549 and PC9 cells exposure to 6 Gy dose of irradiation was monitored in response to LY294002 treatment. Data are expressed as the mean ± SD of different groups of cells from three separate experiments. *P < 0.05, **P < 0.01, ***P < 0.001.

Our previous study demonstrated that RAC1 is closely related to radioresistance in patient samples with lung cancer (38). Herein, we found the mRNA expression levels of RAC1 were up-regulated with the increased dose of X-rays (2, 4, 6, and 8 Gy) up to a maximum level at 8 Gy (Figure 2A). The protein expression of RAC1 showed a similar tendency, in which the protein expression of RAC1 was significantly up-regulated at 4, 6, and 8 Gy (Figure 2B). In addition, as shown in Figure 2C, the results of GST-pull down assays showed Rac1 expression and activity was significantly increased after 6 Gy dose of IR in lung cancer cells, suggesting that IR could promote the Rac1 expression and activity. A question is how IR induces Rac1 expression. According to the report that IR could activate the PI3K/AKT signaling pathway, so we next detected the expression of the effector proteins of the PI3K/AKT signaling pathway after IR, such as PI3K, p-AKT, and AKT. As shown in Figure 2D, the immunoblotting results showed that the PI3K and p-AKT were significantly up-regulated with 6 Gy dose of IR in A549 and PC9 cells. It suggested that IR might induce the activation of PI3K/AKT signaling pathway to promote the Rac1 expression. To investigate whether or not the activation of PI3K/AKT signaling pathway could increase the expression of Rac1, we use the class I PI3K inhibitors, LY294002, to treat the A549 and PC9 cells with 6 Gy dose of IR. The western blot results showed that IR could significantly increase the PI3K, p-AKT, AKT, and RAC1, whereas the LY294002 reversed this effect in both A549 and PC9 cells (Figure 2E). It indicated that Rac1 was the target of the PI3K/AKT signaling pathway, the same as the previous study (36). These results indicate that IR increases the expression and activity of Rac1 via activating the PI3K/AKT signaling pathway.

### RAC1 Promotes Radioresistance, Invasion and Migration in Lung Cancer Cells

**Figure 3:**
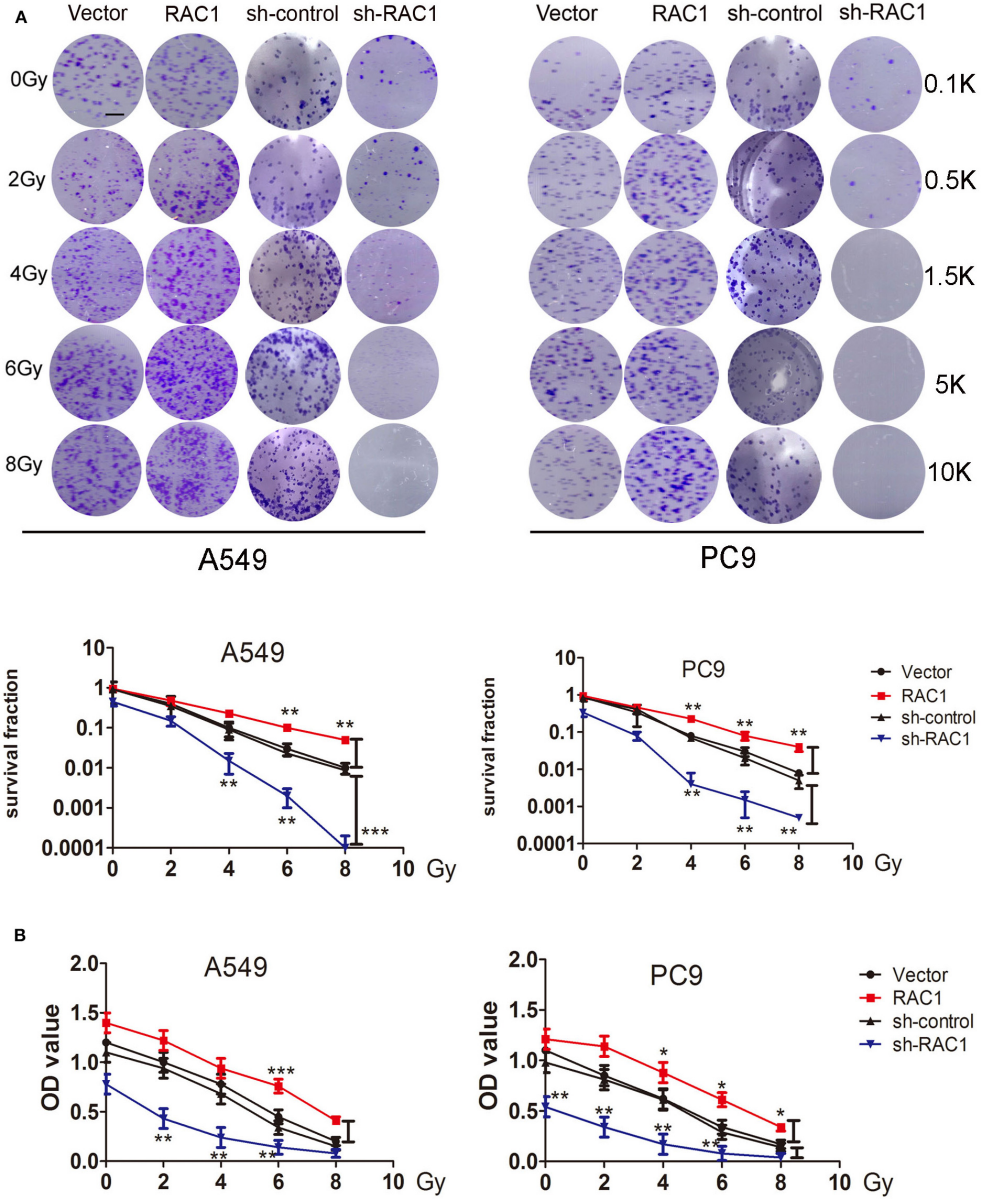
RAC1 inhibition increased the radio-sensitivity for lung cancer cells. **(A)** RAC1-overexpressing A549 and PC9 cells exposure to 0, 2, 4, 6, 8 Gy dose of irradiation promoted cellular radioresistance, and RAC1 silenced A549 and PC9 cells exposure to 0, 2, 4, 6, 8 Gy dose of irradiation attenuated cellular radioresistance under as determined by the clonogenic assay. (Down panel) Dose-survival curve derived from the results of clonogenic assay for A549 and PC9 cells. SF = colonies/(inoculation cells × survival rate). SF represents the sensitivity of cells to RAC1 expression exposed to IR. These numbers (0.1, 0.5, 1.5, 5, and 10 k) represent the number of seeded cells (100, 500, 1,500, 5,000, and 10,000). **(B)** Cell proliferation ability of A549 and PC9 cells within indicated group were detected by CCK-8 assay. Data are expressed as the mean ± SD of different groups of cells from three separate experiments. *P < 0.05, **P < 0.01, ***P < 0.001.

Previous study suggested that up-regulated RAC1 by IR may promote carcinogensis of lung cancer. To assess the effects of RAC1 on the resistance of lung cancer cells to IR, cells were stably transfected with RAC1 (Figure 1A), and then exposed to IR before the survival fraction was analyzed using a colony-forming assay and cell proliferation using CCK-8 assay. The results demonstrated that RAC1 increased survival capacity of A549 and PC9 cells after IR at 0, 2, 4, 6, and 8 Gy (Figure 3A). In contrast, silencing of RAC1 by sh-RAC1 contributed to the sensitivity of lung cancer cells to IR; cells transfected with sh-RAC1 also displayed decreased survival capacity by evaluating survival fraction using a colony-formation assay (Figure 3A). Furthermore, a decrease of cell proliferation was found in A549 and PC9 cells with sh-RAC1 (Figure 3B).

**Figure 4:**
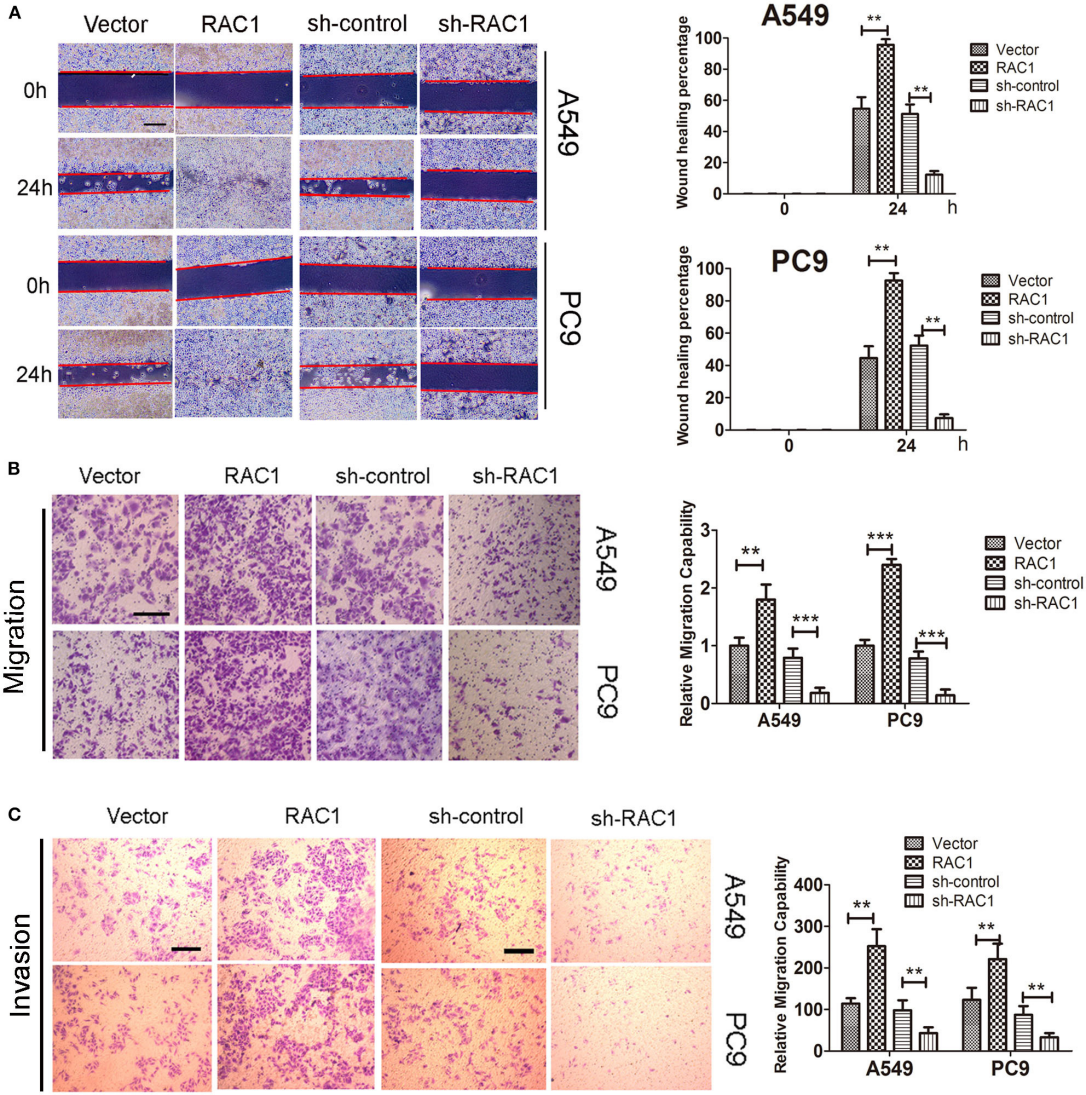
The effects of RAC1 expression on irradiation-induced migration and invasion *in vitro*. A549 and PC9 cells were exposed to 6 Gy doses of IR in the presence or absence of RAC1 expression. **(A)** The influence of RAC1 expression on lung cancer cell migration was determined by wound healing assay in A549 and PC9 cells. Scale bar is 100 μm. **(B)** The effects of RAC1 expression on cell migration detected by Transwell assay, scale bar is 50 μm. **(C)** The effects of RAC1 expression on cell invasion detected by Transwell assay, scale bar is 50 μm. Data are expressed as the mean ± SD of different groups of cells from three separate experiments. **P < 0.01, ***P < 0.001.

We then investigated whether effects of IR on invasion and migration were mediated by RAC1 expression in lung cancer cells. IR promoted A549 and PC9 cells invasion and migration, while down-regulation of RAC1 reversed the effects of IR (Figure S2). In addition, wound healing assay, invasion and migration assay showed that RAC1 promoted cells migration after IR at 6 Gy exposures in A549 and PC9 cells (Figures 4A–C). These results indicate that IR induces the radioresistance and promotes the invasion and migration of lung cancer cells mediated by RAC1 levels.

### IR Induces EMT Phenotypes via Regulating RAC1 Signaling Pathway

**Figure 5:**
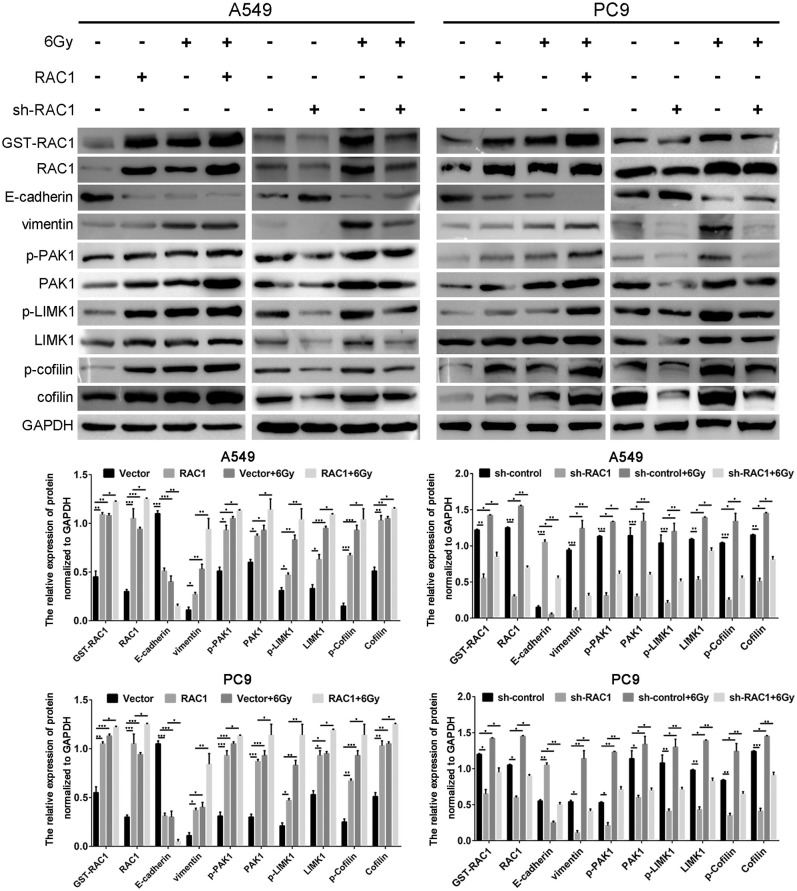
IR induced EMT progression by activating Rac1 signaling pathway in lung cancer cells. A549 and PC9 cells were exposed to 6 Gy radiation in the overexpression and the absence of Rac1 expression. The expression of EMT-related markers, Rac1 expression and activity, and Rac1 signaling pathway related markers (indicated in the figure) was monitored in response to IR treatment. Data are expressed as the mean ± SD of different groups of cells from three separate experiments. *P <0.05, **P < 0.01, ***P < 0.001.

Epithelial-mesenchymal transition (EMT) involves dramatic reorganization of the cytoskeleton, which is a crucial initiating step in tumor invasion and metastasis. IR induces EMT via down-regulating expression of epithelial marker (E-cadherin) and increasing mesenchymal marker expression (vimentin). Herein, we confirmed that IR induced EMT via down-regulating expression of E-cadherin and increasing vimentin expression in A549 and PC9 cells with both the knockdown and overexpression of RAC1 on EMT in IR-treated and untreated cells (Figure 5). Subsequently, further to verify whether RAC1 signaling pathway was involved in the IR-triggered EMT in lung cancer cells, we detected the expression of RAC1 signaling pathway molecules in both the knockdown and overexpression of RAC1 on EMT in IR -treated and untreated cells. As exhibited in Figure 5, RAC1 overexpression led to the up-regulation of GST-RAC1, RAC1, PAK1, p-PAK1, LIMK1, p-LIMK1, Cofilin, and p-Cofilin in A549 and PC9 cells, while the opposite pattern of these genes was found in the A549 and PC9 cells after Rac1 knockdown. IR significantly promoted the expression of GST-RAC1, RAC1, PAK1, p-PAK1, LIMK1, p-LIMK1, Cofilin, and p-Cofilin in the cells treated with IR. The effect of IR on these EMT related markers and molecular markers of RAC1 signaling pathway could be partly augmented by RAC1 overexpression and reversed by RAC1 knockdown (Figure 5). Therefore, these data suggest that RAC1 signaling pathway is involved in the course of IR-induced EMT, and RAC1 is a potential target molecule for the inhibitory effects of IR on lung cancer cell EMT.

### RAC1 Regulates Radioresistance of Transplanted Tumors in Nude Mice

**Figure 6:**
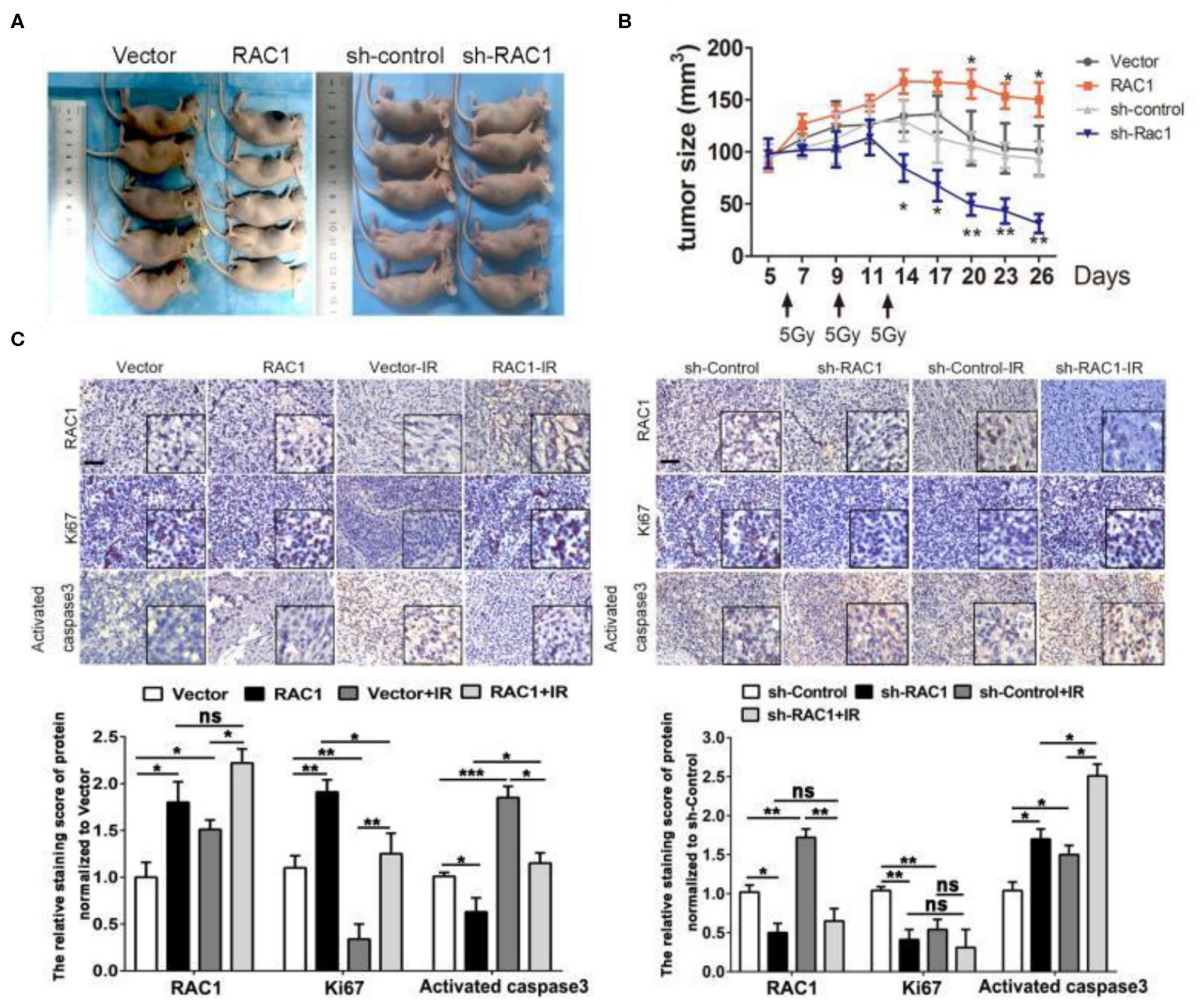
The effects of RAC1 expression in radiotherapy *in vivo*. **(A)** Representative photo of residual tumor of after 3*5 Gy dose of irradiation. **(B)** The time course of growth of Vector, RAC1, sh-control and sh-RAC1 xenograft tumors with or without IR treatment. **(C)** IHC staining showed an elevated Ki67 expression and upregulation of RAC1 in RAC1 overexpression xenograft tumor, which represents the radioresistant process in RAC1 overexpressing cells *in vivo*. Right panel showed a decreased Ki67 expression and downregulation of RAC1 in silencing RAC1 xenograft tumor, which represents the radiosensitivity process in sh-RAC1 cells *in vivo*. Down panel showed the quantification of scoring of immunostaining in RAC1 overexpression/silencing xenograft. Scale bar 100 μm. Data are expressed as the mean ± SD of different groups of cells from three separate experiments. *P < 0.05, **P < 0.01, ***P < 0.001.

We next investigated whether RAC1 could affect NSCLC cellular response to IR *in vivo*. RAC1-overexpressing A549 cells or sh-RAC1 cells and their control cells were inoculated into 4–6 weeks of female nude mice. When tumors reached a size of at least 120 mm^3^, the xenografts were irradiated at a dose of 15 Gy (5 Gy × 3 fractions). Consistent with the *in vitro* results, RAC1 significantly enhanced tumor xenograft growth treated with IR (Figures 6A,B). In contrast, sh-RAC1 group showed a decreased size of tumors responding to IR, but sh-RAC1 significantly inhibited the tumor xenograft growth treated with IR (Figures 6A,B). The expressions of RAC1, Ki-67, and activated caspase-3 were detected in the xenograft tumor tissues of A549 cells in mice (Figure 6C). Increased Ki67 and RAC1 expression and decreased activated caspase-3 levels were observed in the RAC1 over-expression group compared to the Vector group, while this effect was significantly reversed in group with IR treatment (Figure 6C). In addition, the reduced the levels of Ki67 and RAC1, and raised activated caspase-3 were observed in sh-RAC1 group, but IR could enhance the effect (Figure 6C). These results *in vivo* and *in vitro* suggest RAC1 regulates radio-resistance in lung cancer.

## Discussion

RAC1 is constitutively activated in a great majority of lung cancer and contributes critically to the development and progression of lung cancer via EMT (40). In the present study, we observed a striking up-regulation of RAC1 level in NSCLC cells. The RAC1 signaling pathway is required for transformation mediated by the Ras oncogene (41). The RAC1 pathway promotes transformation, protects against apoptosis, and promotes motility and invasion (42–44). It has been reported that Rac1 is the target molecule of the PI3K/AKT pathway. When the PI3K/AKT pathway was activated, the expression and biological activity of Rac1 were significantly up-regulated (36). Meanwhile, RAC1b expression is positively associated with increased growth and chemo-resistance via enhancing NF-κB activity and then activating NF-κB signaling in colorectal cancer (45). In this report, we provide evidence that RAC1 promoted cell proliferation and colony formation in lung cancer cells; silencing of RAC1 expression inhibited pro-survival signal of lung cancer *in vivo* and *in vitro*. These evidences strongly indicate that RAC1 contributes to lung cancer progression and maybe a useful prognostic biomarker of lung cancer.

IR plays a crucial role in cancer treatment; however, radio-resistance leads to distant metastases in patients with radiotherapy (46). Accumulated evidence has demonstrated the important role of EMT in metastasis (47). In this study, we found that IR time-dependently induced EMT in A549 and PC9 cells as indicated by the expression of EMT marker proteins. In lung cancer cells, IR can induce the EMT via activating TGF-β signaling pathway. In this report, we found that IR could induce the up-regulation of RAC1 expression and activity via activating the PI3K/AKT signaling pathway (36, 48–50). Interestingly, IR-induced EMT is regulated by RAC1, a member of the Rho family of small guanosine triphosphatases (GTPases) that has been shown to play a critical role in cytoskeleton reorganization, cell migration and cell survival (51, 52). Thus, we raised the possibility that RAC1 might be an important regulator in the process of IR-induced EMT in lung cancer.

Recently, it has been identified that the RAC1 signaling pathway is an important regulator of the response of breast and pancreatic cancer cells to IR (32, 33). E.g., RAC1 is activated by IR and the inhibition of RAC1 abrogates G2 checkpoint activation and cell survival following IR in breast cancer cells (32, 53). Biochemical analyses indicate that RAC1 inhibition alone could reduce phosphorylation of ERK1/2 and IκBα, as well as levels of anti-apoptotic proteins Bcl-xL and Mcl-1L in the non-irradiated HFR-selected cells (32). The similar role played by RAC1 was observed in pancreatic cancer cells (32). Pancreatic cancer cells are notoriously resistant to the toxicity of radiation therapy (54, 55). In this article, we uncovered inhibition of RAC1 in lung cancer cells is sufficient to abrogate the IR-induced and RAC1-mediated tumor migration and invasion, as evidenced by cell proliferation, colony formation, and Transwell assay. In addition, activation of RAC1 accelerates this process induced by IR. These findings suggest RAC1 contributes to the regulation of radioresistance in lung cancer cells. The inhibition of RAC1 also abrogates the IR-induced mesenchymal markers vimetin and Snail, which plays an important role in EMT. These results reveal that RAC1 protect lung cancer cells from the cytotoxic effects in response to IR. These data raises the possibility that the intrinsic radioresistance of lung cancer cells might be a consequence of the constitutive activation of the RAC1 pathway in this disease.

The effect of the alterations on radiosensitivity caused by RAC1 inhibition is markedly increased in radiosensitivity of pancreatic cancer cells, as demonstrated by caspase-3 activation (32). In this study, we also found that overexpression of Rac1 significantly promoted the migration and invasion, and radioresistance of lung cancer cells, whereas the knockdown of Rac1 markedly inhibited these capabilities of lung cancer cells *in vivo* and *in vitro*. Further studies will be needed to test this possibility and to decipher the mechanisms involved. IR can also simultaneously induce multiple signaling pathways that promote cell survival, such as AKT, ATM/ATR, and ERK cascades (56). It is well-known that the pro-survival signaling pathways generally lead to suppression of apoptosis, activation of cell cycle checkpoint and initiation of DNA repair in cancer cells. By reducing the magnitude of IR-induced cytotoxicity and these pro-survival pathways promote radioresistance in cancer cells. Here, we uncover a novel function for RAC1 signaling in the survival of lung cancer cells in response to IR, suggesting the oncogenetic roles of RAC1 in radioresistance.

Our previous study found that RAC1 could significantly induce the EMT of colon cancer cells, which may be related to the positive regulation of Rac1/PAK1/LIMK1/Cofilins signaling pathway (36). In the present study, we found that IR promoted lung cancer cell EMT in the process, accompanied with an up-regulation of RAC1, PAK1, p-PAK1, LIMK1, p-LIMK1, Cofilin, p-Cofilin in these cells. Silencing Rac1 significantly inhibited the EMT phenotype in lung cancer cells, accompanied with a significant down-regulation of RAC1, PAK1, p-PAK1, LIMK1, p-LIMK1, Cofilin, and p-Cofilin in lung cancer cells. Additionally, IR could enhance the expression and activation of RAC1, positively associated with the up-regulation of PAK1, p-PAK1, LIMK1, p-LIMK1, Cofilin and p-Cofilin. Besides, the effect of IR on EMT and these molecular markers of Rac1 signaling pathway could be partly reversed by Rac1 knockdown, but augmented by Rac1 overexpression. Thus, a better understanding of the mechanisms that promote survival following IR would potentially allow for the identification of novel therapeutic targets to be explored for radiosensitization of lung cancer cells.

## Data Availability Statement

The datasets generated for this study are available on request to the corresponding author.

## Ethics Statement

The animal study was reviewed and approved by the Institutional Animal Care and Use Committee of the Hunan Cancer Hospital and The Affiliated Cancer Hospital of Xiangya School of Medicine, Central South University.

## Author Contributions

YZ, QL, ST, HeW, PY, and LX designed the experiments and wrote the paper. ST, HeW, PY, LX, LO, JLin, JLia, YT, LT, SR, and BZ conducted the experiments. JLin, YH, HuW, YS, QP, MS and JLia collected the clinical data and sample disposal. All authors read and approved the final manuscript.

## Conflict of Interest

The authors declare that the research was conducted in the absence of any commercial or financial relationships that could be construed as a potential conflict of interest.
