# Corrigendum: RAC1 Involves in the Radioresistance by Mediating Epithelial-Mesenchymal Transition in Lung Cancer

**DOI:** 10.3389/fonc.2020.01106

**Published:** 2020-07-14

**Authors:** Shiming Tan, Pin Yi, Heran Wang, Longzheng Xia, Yaqian Han, Hui Wang, Biao Zeng, Lu Tang, Qing Pan, Yutong Tian, Shan Rao, Linda Oyang, Jiaxin Liang, Jinguan Lin, Min Su, Yingrui Shi, Qianjin Liao, Yujuan Zhou

**Affiliations:** ^1^Hunan Key Laboratory of Translational Radiation Oncology, Hunan Cancer Hospital and The Affiliated Cancer Hospital of Xiangya School of Medicine, Central South University, Changsha, China; ^2^Hunan Cancer Hospital, University of South China, Hengyang, China; ^3^Hepatology Unit, Department of Infectious Disease, Nanfang Hospital, Southern Medical University, Guangzhou, China

**Keywords:** RAC1, lung cancer, radioresistance, epithelial-to-mesenchymal transition, metastasis

The authors regret that there was an error in Figure 6 due to incorrect image editing in Figure 6C. The correct Figure 6 appears below.

**FIGURE 6:**
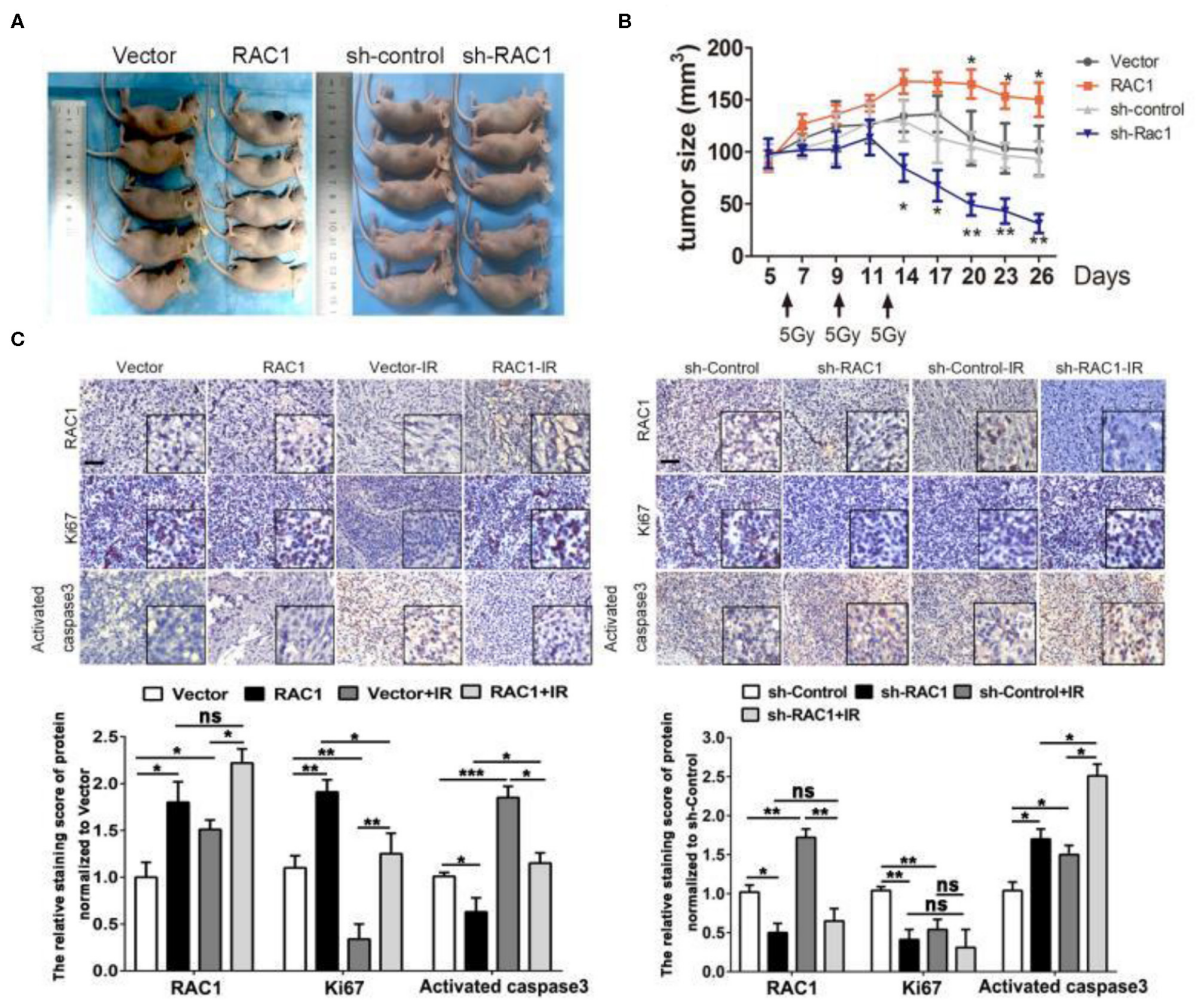
The effects of RAC1 expression in radiotherapy *in vivo*. **(A)** Representative photo of residual tumor of after 3*5 Gy dose of irradiation. **(B)** The time course of growth of Vector, RAC1, sh-control and sh-RAC1 xenograft tumors with or without IR treatment. **(C)** IHC staining showed an elevated Ki67 expression and upregulation of RAC1 in RAC1 overexpression xenograft tumor, which represents the radioresistant process in RAC1 overexpressing cells *in vivo*. Right panel showed a decreased Ki67 expression and downregulation of RAC1 in silencing RAC1 xenograft tumor, which represents the radiosensitivity process in sh-RAC1 cells *in vivo*. Down panel showed the quantification of scoring of immunostaining in RAC1 overexpression/silencing xenograft. Scale bar 100 μm. Data are expressed as the mean ± SD of different groups of cells from three separate experiments. *P < 0.05, **P < 0.01, ***P < 0.001.

The authors apologize for this error and state that this does not change the scientific conclusions of the article in any way. The original article has been updated.

