# Supplementary material for: RAC1 Involves in the Radioresistance by Mediating Epithelial-Mesenchymal Transition in Lung Cancer
Source: Front Oncol. 2020 Apr 28;10:649. doi: 10.3389/fonc.2020.00649 (PMC7198748; doi:10.3389/fonc.2020.00649)
Supplement: Supplementary file 1 [file Data_Sheet_1.PDF]

Supplementary data

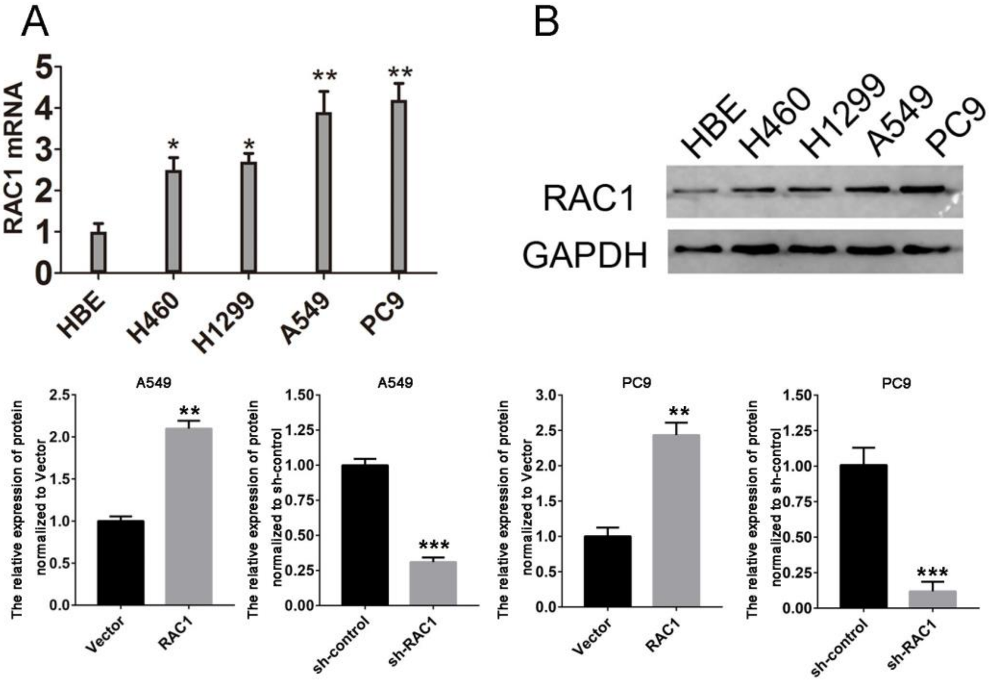

Fig. S1 The expression of RAC1 in normal lung cell and NSCLC cell lines. (**A**) Quantitative analysis of Rac1 mRNA transcripts in normal lung cell and NSCLC cell lines by qRT-PCR. (**B**) Rac1 protein levels in normal lung cell and NSCLC cell lines determined by Western blotting. (**C**) The quantification analysis of RAC1 protein in A549 cell with stably RAC1 overexpression/knockdown and PC9 cells with stably RAC1 overexpression/knockdown. Data are expressed as the mean ± SD of different groups of cells from three separate experiments. $^*P < 0.05$, $^{**}P < 0.01$, $^{***}P < 0.001$.

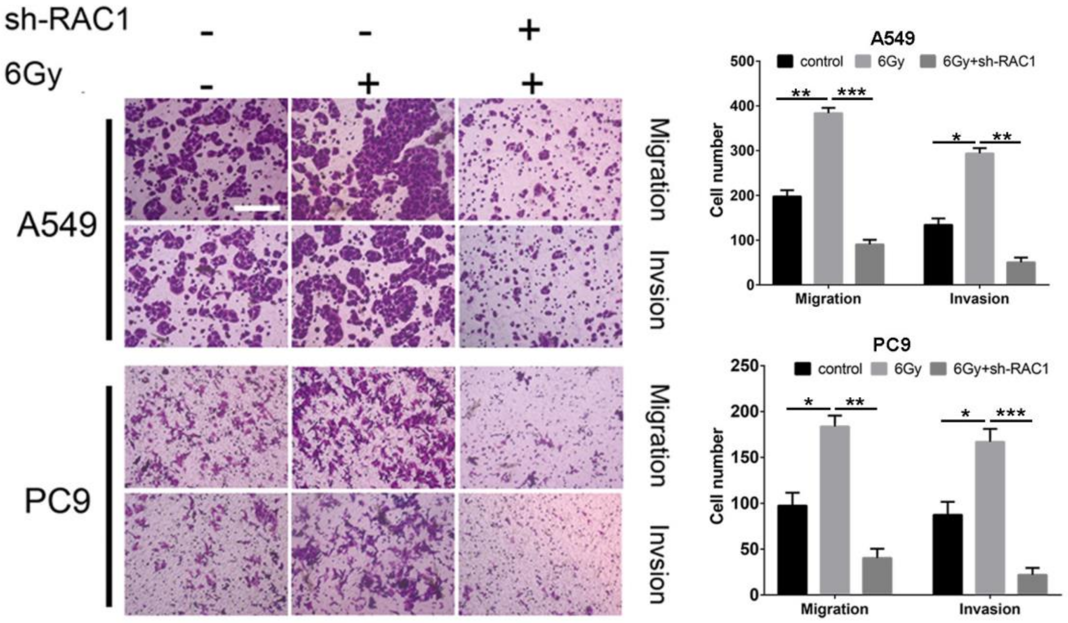

Fig. S2 The effects of RAC1 on IR-induced invasive phenotype in NSCLC cell lines. Transwell assay to detect the effects of RAC1 expression on cell migration, scale bar is 50 μm, Data are expressed as the mean ± SD of different groups of cells from three separate experiments. *$P < 0.05$, **$P < 0.01$, ***$P < 0.001$.
